# Single-dose intraoperative radiotherapy during lumpectomy for breast cancer: an innovative patient-centred treatment

**DOI:** 10.1038/s41416-020-01233-5

**Published:** 2021-02-02

**Authors:** Jayant S. Vaidya, Max Bulsara, Michael Baum, Jeffrey S. Tobias, on behalf of the TARGIT-A trial authors, Frederik Wenz, Samuele Massarut, Steffi Pigorsch, Michael Alvarado, Michael Douek, Christobel Saunders, Henrik L. Flyger, Wolfgang Eiermann, Chris Brew-Graves, Norman R. Williams, Ingrid Potyka, Nicholas Roberts, Marcelle Bernstein, Douglas Brown, Elena Sperk, Siobhan Laws, Marc Sütterlin, Tammy Corica, Steinar Lundgren, Dennis Holmes, Lorenzo Vinante, Fernando Bozza, Montserrat Pazos, Magali Le Blanc-Onfroy, Günther Gruber, Wojciech Polkowski, Konstantin J. Dedes, Marcus Niewald, Jens Blohmer, David McCready, Richard Hoefer, Pond Kelemen, Gloria Petralia, Mary Falzon, David J. Joseph

**Affiliations:** 1 Division of Surgery and Interventional Science, University College London, London, UK; 2 Department of Biostatistics, University of Notre Dame, Fremantle, WA Australia; 3 Department of Clinical Oncology, University College London Hospitals, London, UK; 4 Department of Radiation Oncology, University Medical Centre Mannheim, Medical Faculty Mannheim, Heidelberg University, Heidelberg, Germany; 5 Department of Surgery, Centro di Riferimento Oncologico di Aviano (CRO) IRCCS, Aviano, Italy; 6 Department of Gynaecology and Obstetrics, Red Cross Hospital, Technical University of Munich, Munich, Germany; 7 Department of Surgery, University of California, San Francisco, CA USA; 8 Nuffield Department of Surgical Sciences, University of Oxford, Oxford, UK; 9 School of Surgery, University of Western Australia, Crawley, WA Australia; 10 Department of Breast Surgery, University of Copenhagen, Copenhagen, Denmark; 11 London, UK; 12 Department of Surgery, Ninewells Hospital, Dundee, UK; 13 Department of Surgery, Royal Hampshire County Hospital, Winchester, UK; 14 Department of Gynaecology and Obstetrics, University Medical Centre Mannheim, Medical Faculty Mannheim, Heidelberg University, Heidelberg, Germany; 15 Department of Radiation Oncology, Sir Charles Gairdner Hospital, Perth, WA Australia; 16 Department of Oncology, St Olav’s University Hospital, Trondheim, Norway; 17 Department of Clinical and Molecular Medicine, Norwegian University of Science and Technology, Trondheim, Norway; 18 University of Southern California, John Wayne Cancer Institute & Helen Rey Breast Cancer Foundation, Los Angeles, CA USA; 19 Department of Radiation Oncology, Centro di Riferimento Oncologico di Aviano (CRO) IRCCS, Aviano, Italy; 20 Veneto Institute of Oncology IOV - IRCCS, Padua, Italy; 21 Department of Radiation Oncology, University Hospital, The Ludwig Maximilian University of Munich, Munich, Germany; 22 Radiotherapy-Oncology, Western Cancer Institute, Nantes, France; 23 Breast Centre Seefeld, Zurich, Switzerland; 24 Department of Surgical Oncology, Medical University of Lublin, Lublin, Poland; 25 Breast Centre, University Hospital Zurich, Zurich, Switzerland; 26 Saarland University Medical Center, Homberg, Germany; 27 Sankt Gertrauden Hospital, Charité, Medical University of Berlin, Berlin, Germany; 28 Princess Margaret Cancer Centre, Toronto, ON Canada; 29 Sentara Surgery Specialists, Hampton, VA USA; 30 Ashikari Breast Center, New York Medical College, New York, NY USA; 31 Department of Surgery, University College London Hospitals, London, UK; 32 Department of Pathology, University College London Hospitals, London, UK

**Keywords:** Breast cancer, Radiotherapy, Surgical oncology

## Abstract

In the randomised TARGIT-A trial, risk-adapted targeted intraoperative radiotherapy (TARGIT-IORT) during lumpectomy was non-inferior to whole-breast external beam radiotherapy, for local recurrence. In the long-term, no difference was found in any breast cancer outcome, whereas there were fewer deaths from non-breast-cancer causes. TARGIT-IORT should be included in pre-operative consultations with eligible patients.

## Main

In 1996, the *British Journal of Cancer* published original work from our group, describing widespread spatial distribution of additional cancer foci in mastectomy specimens of patients who were otherwise suitable for breast conservation.^1^ We proposed that these foci may not be clinically relevant because of local recurrence after breast conservation occurs mainly at the site of primary tumour. The TARGIT-A randomised trial^2^ was firmly rooted in this initial observation, and compared risk-adapted single-dose targeted intraoperative radiotherapy (TARGIT-IORT) given during lumpectomy vs conventional whole-breast external beam radiotherapy (EBRT) in an international randomised non-inferiority trial.

The long-term results of the randomised TARGIT-A trial were recently published.^3^ They confirmed comparable long-term effectiveness of risk-adapted TARGIT-IORT and EBRT in terms of breast cancer control. At 5-years complete follow-up, for the primary outcome of absolute difference in raw local-recurrence rates was 1.16% with the upper 90% confidence limit of 1.99%, confirming non-inferiority at the prespecified margin of 2.5%. With long-term follow-up (median 9 years, maximum 19 years), no statistically significant difference was found in local or distant control of breast cancer, breast-preservation or breast cancer mortality. Deaths from causes other than breast cancer were significantly fewer in the TARGIT-IORT arm—HR 0.59 (0.40–0.86) *P* = 0.005, with 12-year rates being 5.41 vs 9.85%, a reduction of 4.44%.

In this commentary we would like to address a number of critical points.

1. The first of these is to emphasise that TARGIT-A trial was not restricted only to patients with a very low risk of local recurrence. Participants had a much higher risk profile than with other trials of partial breast irradiation (PBI, Table 1^3–11^). These other trials restricted the trial entry much more stringently, only recruiting patients with the best prognostic features. By contrast, a substantial absolute number of patients in TARGIT-A, just like the Fast-Forward trial of shorter-course whole breast radiotherapy (Table 2)^12^ were at higher risk of relapse: 1898 (83%) were younger than 70 years, 366 (16%) had tumours >2 cm in size, 443 (20%) patients had grade 3 cancers, 488 (22%) patients had involved nodes and 426 (19%) had ER- or PgR-negative tumours.
  Similarly, patients in the three main trials comparing radiotherapy vs no-radiotherapy (Table 2—CALBG, BASO-II and PRIME-II),^13–16^ were again very highly selected for their low-recurrence risk. By contrast with TARGIT-A, they were strictly limited to those older than 65 or 70 years, with smaller, lower grade, node negative and ER-positive tumours. Despite this, the 5-year local-recurrence rates with ‘no-radiotherapy’ were 2–3 times higher than those seen with TARGIT-IORT (Table 2).
  For the record, most patients in the TARGIT-A trial who had high-risk features did not receive supplemental EBRT after TARGIT-IORT as part of the risk-adapted approach. For example, supplemental EBRT was not given to 78% of Grade 3, not given to 82% of ER-negative and not given to 63% of node-positive patients. Rather, the decision regarding use of supplemental EBRT was made for the individual patient by the treating multidisciplinary team, particularly bearing in mind the main indications of unexpected lobular cancer and positive margins. We regard this as a more patient-centred approach, which takes account of the individual patient-specific circumstances, including their preferences.
  What does all this add up to? Data from the TARGIT-A trial suggest that PBI using this risk-adapted TARGIT-IORT approach is applicable to a breast cancer population more widely inclusive than those recruited in other PBI or ‘no-radiotherapy’ trials. By having TARGIT-IORT during their lumpectomy, 8 out of 10 patients complete their radiotherapy right away, and the benefits include avoiding repeated hospital visits,^17^ a generally lower toxicity, and an improved quality of life.^18–23^
2. An important statistical point relates to the use of Kaplan–Meier (K–M) curves. These are very informative if properly computed. The first step for estimating the risk of any event (e.g. local recurrence), is to categorise each patient into either having the event or not. The time-to-event is then used to plot a graph. This would work well if everyone’s follow-up was the same and no one died, but this of course is never the case because patients are never recruited all at the same instant in any trial. The K–M model therefore uses a method called ‘censoring’, which means that a patient’s data are used until the point when they were last seen. The assumption is that they are alive after they were last seen and continue to have a risk of having local recurrence. But, sadly, some patients die during follow-up, at which point this assumption is of course no longer true. So, when plotting K–M estimates for local recurrence, one should not categorise patients who have died as ‘censored’. Such a plot *must include death* as an event.^3^ Both the plot and any estimate in which the dead have been censored are set in an imaginary world where there is a continual risk of local recurrence after death. Unfortunately, such graphs have frequently been published and are inevitably misleading to readers.
  Here is an example to make this clearer. Let’s look at the NSABP-B39 data.^24^ Their K–M graph of local recurrence shows that the chance of having local recurrence with PBI at 10 years is 4.6%, therefore 95.4% of patients can be expected to be local-recurrence free. This immediately leads to a paradox because in fact, only 90.6% are alive at 10 years, so how can a larger number of patients (95.4%) be around (alive) to be local-recurrence free? A further example comes from the CALGB trial,^14^ in which over 90% patients are estimated to be alive without local recurrence at 10 years, when in fact only 60% are actually alive. Thus, such a K–M graph allegedly depicting local control over time is misleading.
  For this reason both DATECAN^25^ (European) and STEEP^26^ (American) guidelines, rightly insist that death and local recurrence should both be included as clinical events for assessing local treatments for breast cancer.
  Most importantly of all, patients naturally need to know the local control achieved by any new approach compared with the previous standard, which is precisely provided by the outcome of local-recurrence-free survival.
3. Next, we would like to discuss the persistent finding of fewer non-breast cancer deaths with TARGIT-IORT, compared with whole-breast radiotherapy. The reduction was mainly due to fewer deaths from cardiovascular or lung problems and from other cancers and was not small in magnitude: 41% in relative terms and 4.4% at 12 years in absolute terms.
  Randomisation, especially when the trial size is large, ensures that both known and unknown factors are well balanced. In the TARGIT-A trial, all known prognostic factors^3^ were well balanced, as well as age and body mass index (BMI),^3^ relevant for risks of cardiovascular^27^ and malignant disease.^28^
  This somewhat surprising observation is in fact consistent with the results of meta-analyses of randomised trials comparing partial breast irradiation with whole-breast irradiation.^29,30^ It is well to remember that even modern radiotherapy increases cardiac and lung cancer mortality.^31–35^ This is particularly important in current or ex-smokers,^31^ in which a survival decrement of 6% is estimated over a 30 year period. This detriment is likely to outweigh any possible survival benefit from radiotherapy for these patients with early breast cancer.^36^
  Perhaps even more important nowadays, in patients with screen-detected cancer, where the dangers of overtreatment are now so well recognised,^37^ we argue that it is both logical and in the patient’s interest to use TARGIT-IORT, in order to minimise side effects.
4. Finally, it is obvious that this work has special relevance during the current COVID-19 pandemic during which additional visits for radiotherapy consultations, planning and treatment all raise the risks to a vulnerable population as well as adding to pressures on an overstretched hospital system. TARGIT-IORT could help reduce these risks and save precious resources.

**Table 1:** Modern trials comparing partial breast irradiation with whole breast radiotherapy.

For NSABP-39 overall LR used for balloon. External beam days includes half a day for planning. The very old or small trials with less than 500 patients or those with less than 5-year follow-up—from Leeds (EBRT over 28 days, *n* = 174, published 2005)^38^ and Christie (EBRT 10 days, *n* = 708, published 1995)^39^ both with worse outcome for PBI, Budapest (interstitial wires twice a day over 7 days, *n* = 258, published 2013) with similar outcome for PBI^40^ and trials with no published cancer outcome data^41^ are not included in this table. Table reproduced and slightly modified from Vaidya, J.S., Bulsara, M., Baum, M. et al. Intraoperative radiotherapy for breast cancer: powerful evidence to change practice, *Nature Reviews Clinical Oncology*. 10.1038/s41571-021-00471-7 (2021). Numbers are for patients with invasive breast cancer.

*bkgr* expected background risk in the control arm, *ET* endocrine therapy, *QOL* quality of life.

**Table 2:** Modern trials of no-radiotherapy, the trial of short course whole-breast radiotherapy and the TARGIT-A trial.

	CALGB No RT vs WBRT	BASO 2 No RT vs WBRT	PRIME 2 No RT vs WBRT	FAST-FORWARD WBRT vs shorter WBRT	TARGIT-A trial risk-adapted single-dose TARGIT-IORT vs WBRT
Number for comparison	636	1135	1326	2562	2298
Number at 6-year follow-up	<500	N/A	<600	1025	1967
Age limits	≥70 0% < 70	≥65 0% < 65	≥65 0% < 65	>18 84% < 70	≥45 60% < 65 85% < 70
T Size limits	≤2 cm	≤2 cm	≤3 cm	T1–T3	≤3.5 cm
Grade limits	No info.	Grade 1	Grade 1 or 2, only 2% grade 3	No restriction 28% grade 3	No restriction 20% grade 3
Nodes limits	Negative	Negative	Negative	N0–N1 19% node positive	No restriction 22% node positive
LV invasion	No info.	Negative	Neg if Gr 3	No restriction	No restriction
ER status	Positive	Positive	Positive	No restriction	No restriction
Additional hospital visits	1	1	1	7–15	None in 80% of cases; WBRT recommended in 20%
5-year local-recurrence rates	4 vs 1%	6 vs 2%	4.1 vs 1.3% Difference 2.9% (upper 95%CI 4.8%)	2.1 vs 1.4% (including 7% post-mastectomy radiotherapy) No difference	2.11 vs. 0.95% Non-inferiority confirmed with complete 5-year follow-up Difference 1.16% Upper 90%CI 1.99%
Long-term outcomes, more than 5 years	10-yr OS 67 vs 66%; LR 8 vs 2%; 10-yr LRFS ~53 vs ~61%	10-yr LRFS ~89 vs ~97%	10-yr LR 9.8% vs 0.9%. Binomial 10-year Non-breast cancer deaths 3.9% vs 6.1% and total deaths 13.2% vs 12%	Not available	At median follow-up of 9 years (max 19 years): No difference in local/distant control/breast preservation/breast cancer mortality Significantly fewer deaths from other causes (5.41% vs 9.85% at 12 years)
Significant scattered radiation to vital organs?	No	No	No	Yes	No
Mortality	No difference	No difference	No difference	No difference	Significantly reduced non-BC mortality with TARGIT-IORT No difference in BC mortality
Toxicity in experimental arm	Not reported	Not reported	Not reported	Higher (e.g. breast induration/hardness)	Reduced
Quality of life with experimental treatment	Not reported	Not reported	Higher insomnia No improvement in QOL	Not reported	Improved breast related QOL Improved cosmetic outcome Reduced pain

### Conclusion

Using the approach of risk-adapted TARGIT-IORT in patients with early breast cancer avoids the inconvenience and toxicity of whole-breast radiotherapy in 8 out of every 10 patients. When compared with whole-breast radiotherapy in the randomised TARGIT-A trial, now with long-term follow up, no difference was found for any breast cancer outcomes, but there was a reduction in non-breast cancer mortality with TARGIT-IORT. Previous studies have shown that the other advantages include reduced breast pain, a better quality of life,^18–23^ a cosmetically superior outcome and reduced travelling time for the patient.^17^

Clinicians and patients in 38 countries (260 centres) have been adopting TARGIT-IORT since the publication of the first results, and over 45,000 patients have been treated so far. We believe that the long-term data,^3^ taken together with the many obvious benefits for the patient, provide compelling evidence to roll this out further.

Finally, all doctors in the UK are now obliged to follow the recently published GMC guidelines which underline the essential nature of adequate patient information—i.e. what they can reasonably expect to be told—in order to provide valid consent (https://www.gmc-uk.org/ethical-guidance/ethical-guidance-for-doctors/consent). This powerful principle is now fully enshrined in UK law (Montgomery v Lanarkshire Health Board, 2015).

## Data Availability

Not applicable.
